# Is Estrogen a Missing Culprit in Thyroid Eye Disease? Sex Steroid Hormone Homeostasis Is Key to Other Fibrogenic Autoimmune Diseases – Why Not This One?

**DOI:** 10.3389/fimmu.2022.898138

**Published:** 2022-06-17

**Authors:** Amy M. FitzPatrick

**Affiliations:** Independent Researcher, Los Angeles, CA, United States

**Keywords:** estrogen, thyroid eye disease, TED, Graves’ orbitopathy, estrogen receptor, steroid homeostasis, Graves’ disease, sex steroids

## Abstract

Sex bias in autoimmune disease (AID) prevalence is known, but the role of estrogen in disease progression is more complex. Estrogen can even be protective in some AIDs; but in systemic lupus erythematosus (SLE), rheumatoid arthritis (RA), and systemic sclerosis (SSc), estrogen, its metabolites, and its receptors have been demonstrated to play critical, localized inflammatory roles. Estrogen is instrumental to the fibrosis seen in RA, SLE, SSc and other disease states, including breast cancer and uterine leiomyomas. Fibrotic diseases tend to share a common pattern in which lymphocyte–monocyte interactions generate cytokines which stimulate the deposition of fibrogenic connective tissue. RA, SLE, SSc and thyroid eye disease (TED) have very similar inflammatory and fibrotic patterns—from pathways to tissue type. The thorough investigations that demonstrated estrogen’s role in the pathology of RA, SLE, and SSc could, and possibly should, be carried out in TED. One might even expect to find an even greater role for estrogen, and sex steroid homeostasis in TED, given that TED is typically sequalae to Graves’ disease (GD), or Hashimoto’s disease (HD), and these are endocrine disorders that can create considerable sex steroid hormone dysregulation. This paper highlights the pathophysiology similarities in 4 AIDs, examines the evidence of sex steroid mediated pathology across 3 AIDs and offers a case study and speculation on how this may be germane to TED.

## Introduction

Though a fair portion of the inflammatory cascade in thyroid eye disease (TED) is now well characterized, some key aspects of the disease remain enigmatic. For example, why do orbital fibroblasts in patients with TED express insulin-like growth factor-1 receptor (IGF-1R) at higher levels than people without TED? Why does only a subset of patients with GD or HD manifest TED? Some think it is possible that all GD patients do get TED, but it remains subclinical or very mild in most patients. The question would still remain—what keeps those TED cases subclinical? Furthermore, what, precisely, is the effect of euthyroid status on TED? Anti-thyroid medications, used to achieve euthyroid status, such as methimazole affect T4/T3 synthesis but their impact on the immune system remains to be defined. Therefore, it seems unlikely that the drug itself protects against TED. In fact, someone can achieve euthyroid status with methimazole but have no reduction in levels of thyroid autoantibody. Autoantibody levels are also not a direct predictor of TED. It is possible for a patient to have Graves’ disease with a high TSI (thyroid stimulating immunoglobulin) and not to have TED (1, 2). What prevents TSI from antagonizing the orbital TSHRs in these cases? As with any autoimmune disease, there may be multiple answers to that question. But the possible interaction of the endocrine system with the immune system is not yet represented in the TED literature. This is curious because that interaction has been examined in a number of other AIDs – and since Graves’ and Hashimoto’s diseases disrupt endocrine homeostasis, the endocrine-immune interaction in these diseases could be ripe for pathology.

## TED Pathophysiology

Orbital fibroblasts in patients with TED express both thyrotropin receptor (TSHR) and IGF-1R at higher levels than found in fibroblasts of healthy controls. In the case of Graves’ disease, thyroid autoantibodies bind to TSHR, the main autoantigen in TED (3). TSHRs then complex to IGF-1Rs, *via* β-arrestin, beginning the immune activation (2, 4). Lymphocytes migrate to the orbit. B cells are activated *via* their recognition of TSHR, and *via* the CD40:CD40L molecular bridge between B cell and T cell surfaces. B cell proliferation and differentiation ensues. Cytokine releasing T cells are activated, producing adhesion molecules, and facilitating further traffic into the orbit. Activated orbital fibroblasts proliferate, secrete hyaluronan and pro-inflammatory cytokines including IL-1β, IL-1α, IL-6, IL-8, macrophage chemoattractant protein-1 (MCP-1) and transforming growth factor-β (TGF- β). This cascade perpetuates orbital inflammation and facilitates the fibroblast differentiation into adipocytes and myofibroblasts, expanding adipose tissue and fluid accumulation between the muscle fibers. Ultimately, this process increases the volume of the orbital tissues, remodels the orbit, and leads to the characteristic proptosis and congestive features of TED (2, 3, 5).

**Figure 1:**
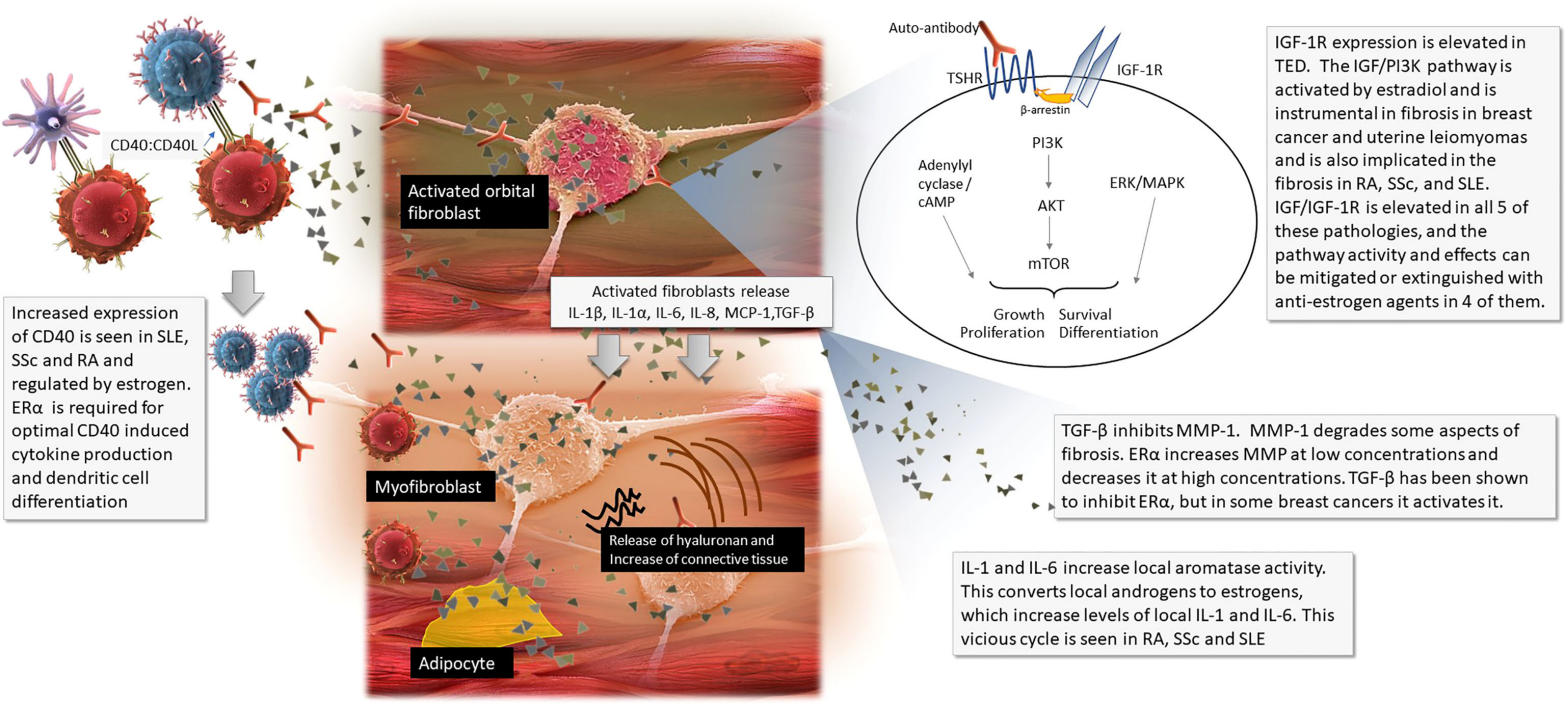
The pathophysiology of thyroid eye disease with callouts of where sex steroids influence other diseases.

Typical of fibrosis, tissue proliferation in TED is mediated *via* the phosphoinositide-3 kinase/AKT/mammalian target of rapamycin (PI3K/AKT/mTOR), adenylyl cyclase/cyclic adenosine monophosphate (cAMP) and MAPK/ERK pathways (**Figure 1** **)** (3, 6, 7).

Clinical manifestations of this pathophysiology include proptosis, double vision, edema around the orbit and in the eyelids, festoons, eyelid retraction, corneal lacerations, pain and increase in buccal fat. The initial inflammatory and progressive phase typically stops after two years and is followed by a stable fibrotic stage (1, 8). Most cases of TED are mild or self-limiting, but up to ~30% require treatment, usually immunosuppressive (9). Multiple surgeries may also be required and in severe cases, TED can be sight threatening (10). A targeted monoclonal antibody antagonist of IGF-1R is now approved by the FDA and has demonstrated efficacy in the treatment of moderate to severe TED (8). The reduction in the patient’s quality of life has been measured as being on par with cancer (11).

The onset of TED can vary but tends to occur within 18 months of a diagnosis with uncontrolled Graves’ disease. It can also occur in patients with Hashimoto’s disease, and rarely even in people with no thyroidopathy (12).

## Epidemiology of TED

The incidence of TED is 16 per 100,000 females and 2.9 per 100,000 males and is found across ethnicities. Like most autoimmunity, the preponderance is higher in women, but as seen in SSc, in severe cases, the ratio reverses to 1:4 females to males (13, 14). The pooled prevalence of TED that occurs in Graves’ disease is 40% (15).

## Similarities in Pathophysiology of Three Model Autoimmune Diseases

Fibroblasts overactivation and aberrant differentiation and proliferation are central to TED pathophysiology. Under physiologic conditions, fibroblasts are sentinels that maintain tissues homeostasis and are therefore flexible and responsive to stimuli and their environment. Reductively speaking, fibrotic disease is essentially aberrant or nonterminating wound healing (6, 16).

RA, SLE and SSc have several similarities to TED pathogenesis. They occur in fibroblast-type tissues and can result in a type of fibrosis. IGF is thought to mediate this fibrosis (17–20). These three AIDs also have other similarities to TED, such as overexpression of CD40 causing issues with the CD40:CD40L molecular bridge; the involve several of the same pro-inflammatory cytokines including IL-1β, IL-1α, IL-6; and the PI3K/AKT/mTOR proliferative pathways. The unusual, characteristic two-year burnout seen in TED is also seen in SSc. Estrogen has been shown to play a pivotal role in the each of these inflammatory and fibrotic pathologies in RA, SSc and SLE. A summary of these similarities can be seen in **Table 1** and **Figure 1** (3, 21–26). Though there are differences among them, given these similarities and the fact that fibrosis and inflammation can have similar pathology across many disease types and are both known to be modulated by estrogen, it seems worth investigating if estrogen, and sex steroid homeostasis, is also instrumental in TED (7, 27).

**Table 1:** Similar pathogenic pathways in 4 autoimmune diseases.

Pathophysiology Component	RA	SSc	SLE	TED
**Monocyte driven fibrosis**	✓	✓	✓	✓
**Occur in fibroblasts or fibroblast type tissue**	✓	✓	✓	✓
**Activated fibroblasts capable of migrating and creating new disease sites**	✓	✓	✓	✓
**Identical key inflammatory pathways**	✓	✓	✓	✓
**Proliferation driven by AKT/mTOR molecular pathways**	✓	✓	✓	✓
**Two-year “burnout” or decrease in disease activity**		✓		✓
**Demonstrated role of IGF and/or IGF-1R in pathophysiology**	✓	✓	✓	✓
**Demonstrated pivotal role of estrogen and estrogen metabolites in pathophysiology**	✓	✓	✓	**?**
**Demonstrated role of estrogen receptors in pathophysiology or protection from pathology**	✓	✓	✓	**?**
**Blunting of *in vitro* pathology with anti-estrogen agents**	✓	✓	✓	**?**
**Blunting of *in vitro* pathology with anti-inflammatory estrogen metabolites**	✓	✓	✓	**?**

Check mark, evidence exists in the literature; question mark, no evidence exists yet.

## Sex Steroid Hormones and the Immune System

Most cells of the innate and adaptive immune system express estrogen receptors, and the endocrine system exerts manifold regulation on the immune cells. This interdependency has been investigated for decades and has yielded many interesting observations about the endocrine system as much as the immune. For example, progesterone receptors are not seen in resting states of monocytes but do appear in pregnancy. You cannot always mimic estradiol effects *in vitro* which are seen *in vivo*, so there may be temporal aspects that vary. Additionally, hormone receptors are found at different concentrations on different types of immune cells, illustrating flexibility in regulation (28–31).

## Estrogen Can Be Anti-Inflammatory or Inflammatory

Estrogen’s impact on the immune system depends on the estrogen receptor type, tissue type, cellular conditions, concentrations of estrogen, concentrations of it relative to other hormones, estrogen metabolites, and, at least in the case of autoimmunity, it may also depend on polymorphisms of estrogen receptor types (25, 32–35).

### Estrogen Receptors

Estrogen receptor alpha (ERα) and beta (ERβ) are both nuclear receptors that once bound with their ligand estradiol (E2) translocate to the nucleus where they can regulate gene expression or serve as cofactors with other transcription factors, such as NF-κB (36, 37). They have strong and equal affinity to the E2 and also have affinity for downstream estrogen metabolites—which can also be either anti-inflammatory or inflammatory depending on the type and ratio to each other (27, 32, 38).

ERα and ERβ (from genes *ESR1* and *ESR2*, respectively) are ubiquitous and are expressed on most immune (innate and adaptive) cells in addition to a large assortment of other cells and tissues (38, 39). ERα and ERβ can be co-expressed in a cell, and each receptor regulates different genes. They are also found preferentially and in different concentrations in different tissue types (28, 31, 36, 38–40). For example, CD4+ T cells, which are helper cells express greater amounts of ERα than Erβ, and a greater amount of both ERs than peripheral blood monocytes or CD8+ T cells, which are cytotoxic. B cells express more Erβ, than ERα (41). ERs regulation of different genes and expression at different levels may explain cellular and tissue-specific response to estrogen.

In general, estrogen activity mediated *via* ERα is proliferative and anti-apoptotic and has therefore been implicated in many proliferative diseases, such as prostate cancer, breast cancer, and autoimmune disorders (23, 31, 34). ERα has recently been demonstrated promote T cell activation and proliferation in autoimmune disease, and to be instrumental to effector functions and dendritic cell development (42–44). The knockout of ERα in SLE murine models resulted in significantly attenuated disease and prolonged survival (45). Other ERα knock out models have demonstrated the receptor’s involvement in physiological lymphoid organ development. Erlandsson et al., showed ERα was mandatory in male mice for development of full-size thymus and spleen, this was not the case in females, who displayed thymic atrophy mediated by ERβ. Of note to TED, these authors noted that a potential background for their findings may have been the down-regulated activity in the growth hormone/IGF-1 axis in males lacking ERα (46). Sánchez-Maldonado, et al., found that 3 *ESR1* haplotypes had a reduced risk of erosive arthritis, and that both *ESR1* and *ESR2* influence the disease (47). Finally, in a colitis mouse model, ERα deletion in T cells reduced their pathogenic potential *via* increased expression of *Foxp3* (42). When the PI3Kδ pathway is activated in lymphocytes, it inhibits FOXO, when it’s not, FOXO is disinhitbited (48). These are just some examples of the manifold ways ERs participate in immune regulation and its pathology, the literature is replete with other examples, as ERs are ubiquitous.

ERβ has been found frequently to be anti-proliferative, and counters ERα’s anti-apoptotic effects (31, 49). In some tissues, ERβ has been shown to prevent or protect against fibrosis (23, 50, 51). ERβ-selective ligands are being investigated as a therapeutic target in Crohn’s disease and rheumatoid arthritis (49, 52).

A third group of more recently discovered and much faster-acting, membrane-bound G-coupled estrogen receptors are also being investigated for their roles in regulating growth factors in cancer and in the immune system (53–55). G protein-coupled estrogen receptor 1 (GPER1) is a Gs-coupled receptor expressed in innate and adaptive immune cells and can facilitate estrogenic activity independently of the other ERs or with their coordination (55, 56).

### Estrogen Metabolites

Estradiol and estrone (E1, a weaker estrogen found in all women, but in higher quantities in post-menopausal women) both are converted into hydroxylated metabolites that can also bind with strong or weak affinity to ERs. E1 and E2 are hydroxylated at the C2, C4 and C16 positions and these 6 metabolites are named thusly:

**Table:** 

• 2-hydroxyestrone (2OH-E1)	• 2-hydroxyestradiol (2OH-E2)
• 4-hydroxyestrone (4OH-E1) and	• 4-hydroxyestradiol (4OHE2)
• 16α-hydroxyestrone (16αOH-E1)	• 16α-hydroxyestradiol (16αOH-E2).

**Figure 2:**
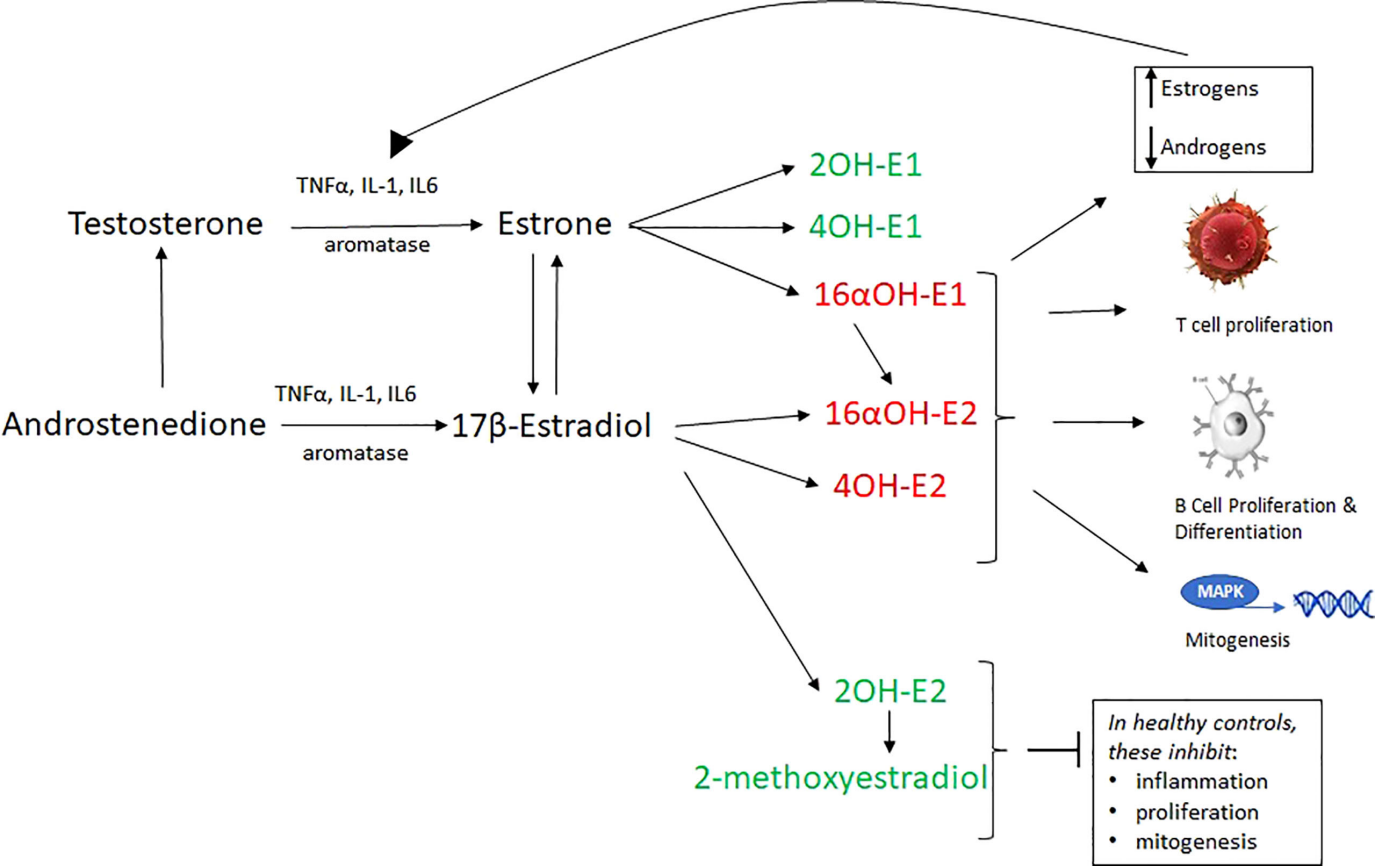
17β-estradiol and estrone, which are derived by the aromatization of testosterone and androstenedione, are converted into 6 hydroxylated metabolites (via cytochrome P450 enzymes) which are inflammatory (red) or non-inflammatory (green). 16α -hydroxylated forms of estrogens covalently bind ERα and allow its proliferative capabilities to flourish. They are important proinflammatory and proproliferative signals. Furthermore, unlike other estrogens, they do not inhibit TNFα. 16αOH-E1 is not only inflammatory, but promotes unscheduled DNA synthesis, mitogenesis, and is a breast cancer tumor initiator. 2-hydroxylated metabolites weakly bind ERα, inhibit the proliferative action of that receptor, and are generally anti-inflammatory, pro-apoptotic. The loss of 2-hydroxylated estrogens (as in RA and SLE) is a proinflammatory signal. Estrogen metabolism in some autoimmune disease favors inflammatory catechol estrogen end products. A vicious cycle wherein IL-6 and other cytokines convert local androgens via aromatase to create more E2, which in turn creates more IL-1 and IL-6. In total, this aberrant estrogen metabolism and increased overall estrogen relative to androgens causes increased T cell proliferation, B cell proliferation, mitogenesis, inflammation and reduced apoptosis.

These catechol estrogens can then be methylated into methyoxyestrogens. In general, the 2OH’s and 4OH-E1 are anti-inflammatory at high concentrations, and the 16αOH’s and 4OH-E2 are inflammatory. 16αOH’s have lower affinity tor ERs than estradiol, but they bond covalently and fail to downregulate the receptor, facilitating proliferation in disease such as cancer and autoimmunity **(** **Figure 2** **)** (33, 35, 57–63).

### Sex Steroid Mediated Immune Homeostasis

#### Progesterone and Testosterone Are Anti-Inflammatory. Low Levels of Either Facilitate Estrogen’s Inflammatory Effects

Progesterone has several anti-inflammatory mechanisms. Like estrogen, it has both membrane and nucelar receptors, but these have not yet been found uniformly across immune cell types as ERs have (64). Progesterone has some capacity to suppress CD4+ T cell proliferation, Th1/Th17 differentiation and effector functions while also being able to enhance Treg differentiation (65–68). Miyaura et al. demonstrated that pregnancy-level progesterone can inhibit differentiation of human Th1 cells, driving them toward an IL-10-secreting phenotype (IL-10 is generally thought to be anti-inflammatory) (65). Additionally, progesterone suppresses IL-6, and reduces IL-6 receptors and suppresses the mTOR pathway which is a proliferative pathway downstream of PI3K used across a wide array of cell types, including lymphocytes, and is instrumental in the fibrosis seen in TED (3, 67, 69–72). Progesterone has also been shown to reduce inflammatory cytokines such as TNFα (67, 73). This partially explains why symptoms in many autoimmune disorders are reduced during the luteal phase of the menstrual cycle (which is dominated by progesterone) and all but eliminated in pregnancy levels of progesterone. Further, these women often see a sharp rebound return of their autoimmune disorder after giving birth, when progesterone plummets.

Testosterone provides a similar inflammatory corrective to estrogen. Like the other steroid hormones, testosterone has influences across immune cell types, cytokines and inflammatory mediators such as C-reactive protein (74). Nasser et al, demonstrated that testosterone therapy was effective compared to placebo in treatment of Crohn’s disease, as measured by Crohn’s Disease Activity Index. The therapy reduced C-reactive protein dramatically and brought leukocytes within normal limits (75). Another example is a murine model that mimics the histology of nonbacterial prostatitis. E2 administration causes dose dependent inflammation in the prostate in castrated mice. E2 exerts this effect on the prostate by stimulating transcript levels of IL-1β, IL-6, macrophage inflammatory protein-2, and iNOS and is accompanied by fibromuscular proliferation, which consisted of fibroblasts, smooth muscle cells, and collagen. The effects increased with time (32, 76).

This homeostatic milieu may be vital to TED, as explained below is the fact that poorly controlled GD or HD facilitates high levels of estrogen and low levels of progesterone and testosterone.

## Estrogen and Estrogen Metabolites in RA, SSc and SLE

Lack of sex steroid homeostasis has been illustrated to impact pathology. Local levels of proinflammatory estrogens relative to androgens are significantly elevated in both male and female patients with RA, SLE and SSc compared to controls (77–79). The cause of this is a vicious cycle wherein local inflammatory cytokines such as TNF, IL-1 and IL-6 induce aromatase activity, converting steroid pre-hormones (DHEA, testosterone and androstenedione) into estrogen. Excess estrogen then stimulates IL-1 and IL-6 production (80–82). The local aromatization decreases plasma androgen levels in patients with RA, SLE, and SSc, compared to controls without disease (77–79). In fact, the lowest levels of serum androgen are seen in women with most active SLE disease (78).

This is further complicated by the estrogen metabolism in these diseases which yields a preponderance of the proinflammatory metabolites 16α-hydroxyestrone and 4-hydroxyestradiol (**Figure 2**) (21, 34, 80, 83). Both of these metabolites have been demonstrated to be mitogenic, angiogenic, and inflammatory in breast cancer, autoimmune disease, pulmonary hypertension, and several other diseases under investigation (57–59, 61, 83–85).

Inflammation and proliferation in these 3 AIDs can be reduced or extinguished *in vitro* using anti-estrogen agents such as tamoxifen or with aromatase inhibitors like anastrozole (33, 42, 80). Of note, progesterone has also been shown to dampen the growth stimulatory effects of estrogen on RA synoviocytes (26, 67).

The inflammatory effects of 16α-hydroxyestrone (16α-OH) are mitigated by another estrogen metabolite, 2OH-estrogen. The urinary 16α-OH/2OH-estrogen ratio is a marker of inflammation used in breast cancer research, and recently in autoimmune disease. Urinary levels of the anti-mitogenic 2OH estrogens are 10 times lower in RA and SLE patients than in healthy controls, making the 16α-OH/2OH estrogen ratio 20 times higher in RA and SLE patients than in healthy controls. This likely contributes to maintenance of the proliferative state in these diseases (33, 83) (**Figure 2**). Why these individuals metabolize estrogen toward an inflammatory profile is unknown, but autoimmune and breast cancer research implicates polymorphic genes for the cytochrome P450 enzymes involved in estrogen metabolism, as well as polymorphisms in ERs (47, 61, 86, 87).

In pre-clinical models, 2-methoxyestradiol (2-ME), has been shown to suppress fibrosis in SSc and to induce apoptosis in diverse tumor types and inhibit experimental autoimmune encephalomyelitis (59, 88, 89). Both 2OH-E2 and 2-ME limit estrogen-driven growth and inflammation in autoimmune disease (21, 33, 85).

Khalkhali-Ellis et al., have also demonstrated the dynamic effects of estrogen and progesterone on fibroblast-like synoviocytes in RA, and showed with ER transfected fibroblast-like synoviocytes, cartilage invasion. This was partially mediated by matrix metalloproteinases, which have complex roles in fibrosis, where they can attenuate or augment the fibrotic pathway (26, 90).

## Estradiol (E2) Modifies Key Inflammatory Mediators and Cell Metabolism in Autoimmune Disease

### E2 Dependent Increase in CD40L Expression Hyperstimulates SLE T Cells, Contributing to the Pathogenesis of SLE

Rider et al., examined T cells in women with SLE vs healthy women. The molecular CD40:40L bridge formed between B and T cells is physiological, but also a point of possible pathology. Increased expression of CD40L can result in spontaneous autoantibody production in autoreactive B cells (91). They bathed SLE T cells and healthy T cells in E2 and had a 3^rd^ group of SLE T cells not bathed in E2 as a second control. They saw a significant increase in CD40L expression (and CD40L mRNA) in the estradiol bathed SLE T cells compared to non-estradiol SLE T cells. These increases could be blocked by an estrogen receptor antagonist. The healthy T cells did not show CD40L expression change with E2 (24). They further demonstrated that E2 is acting *via* ERα, and in SLE T cells activates calcineurin and CD40L, but not in healthy T cells (25). Perhaps a possible explanation is found in the receptors. SLE cells have been shown to express aberrant polymorphic ERα receptors (92).

Further, In SLE, a SERM (selective estrogen receptor modulator) called raloxifene reduced estrogen induced CD40 overexpression on follicular B cells, making them less responsive to T cell co-stimulation (93).

Though it has been previously noted that increased CD40 expression is unique to orbital fibroblasts, significantly increased CD40 expression is seen in RA, SSc and SLE fibroblasts and fibroblast-like synoviocytes (22, 24). In fact, it is also seen autoimmune pulmonary fibrosis, suggesting this is not an uncommon fibrotic phenomenon. However, it cannot be overstated that estrogen mediated immune system influence is tissue and disease specific. E2 actually blocks the induction of CD40 and CD40L expression on cardiovascular endothelial cells and prevents neutrophil adhesion *via* the ERα pathway and prevents cardiac fibrosis *via* the ERβ pathway (50, 51). There is a vast literature on estrogen’s ability to have both anti-inflammatory and inflammatory effects (27).

Looking more directly at the fibrosis in SLE, Corradeti et al. demonstrated metabolism changes conferred due to altered transcription of key genes, as mediated by ERα. Among key results, their findings demonstrate that ERα-mediated metabolism variations exacerbate immune-induced renal damage resulting in increased inflammation and fibrosis. ERα knockout (from both immune and kidney cells) murine models suffered less kidney disease. They also sequenced human patient’s DNA and found similar expression pattern changes (94).

A final inflammatory example is that estrogen induces fibrotic phenotype in SSc, a connective tissue autoimmune disease of unknown origin characterized by fibrosis of the skin, lungs, kidney, and other organs. Focusing on skin fibrosis in SSc, Aida-Yasuoka et al. confirmed the expression ERα and ERβ in primary dermal fibroblasts from twin pairs, six who had SSc, five of whom were healthy. They demonstrate that E2 is an inducer of fibronectin expression, and acting *via* ERα, exerts profibrotic effects. Further, E2 treatment increased further E2 sensitivity by increasing ERα’s. They were able to block these profibrotic effects with an ER antagonist, fulvestrant. Furthermore, ERβ levels were much higher in the healthy twins than in the fibroblasts of the patients with SSc. Aida-Yasuoka et al. suggest ERβ could play a protective role in SSc as has been seen in cardiac fibrosis. Unsurprisingly, the intracellular pathways in the proliferation of fibrosis implicated (via inhibition of each) were P13K and p38 MAPK (23).

## Rationale for Examining the Sex Steroids in TED

When Cutolo and Straub, et al. began examining the exact role of estrogen in the pathophysiology of rheumatoid arthritis, it was speculative. It is clear across autoimmunity that there is a sex bias, but estrogen’s role in direct pathophysiology had not been thoroughly examined. They uncovered a complex and nuanced role for estrogen wherein dose, metabolite, receptor and tissue type made the difference between joint destruction and healthy joint (35, 62). Given the similarity in pathophysiology between RA and SLE, they examined the role of estrogen in this second autoimmune disease – finding it exerted the same influence (34). Then, given the similar fibrogenic pathology, investigators examined estrogen in SSc, discovering, yet again, a salient role (80). I suggest that given the similar fibrogenic and inflammatory cascades, we extend these investigations one disease further – to TED.

Since this would be an entirely new line of inquiry, direct evidence for the role of sex steroids in TED is scant, but the profound sex steroid disruption seen in uncontrolled GD and HD begs the question.

## Autoimmune Thyroid Disease Disrupts Sex Steroid Hormone Homeostasis Toward an Inflammatory Milieu

As outlined above, patients with RA, SSc and SLE have slightly higher levels of serum estrogen to androgens, caused by the local levels of increased aromatization (34, 77). E2 levels in women with hyperthyroidism are estimated to be 2-3-fold higher than normal controls, while progesterone levels are often lower than normal (95, 96). Metabolic clearance rate of E2 is decreased in both genders. Testosterone and androstenedione (androgens) are increased, but so is the conversion ratio of androstenedione to estrone, as well as of testosterone to E2 in hyperthyroid women. Bioavailable testosterone levels are subnormal in hyperthyroid males, while free E2 are elevated—making the free testosterone/free E2 ratio lower in hyperthyroid males compared with normal individuals. Progesterone can be reduced in women in either hyper- or hypothyroidism (97–100) **(** **Table 2** **)**. These ratios engender an inflammatory milieu similar to those seen in RA, SLE and SSc, and indeed more profoundly disrupted. Could TED’s localized inflammatory cascade also have steroid hormones participation, as in these three model diseases?

**Table 2:** Synopsis of hormonal changes in male and female thyrotoxicosis and hypothyroidism.

	Thyrotoxicosis		Hypothyroidism	
	Males	Females	Males	Females
**E2**	N or ↑	↑	N	↓
**Clearance Rate of estrogens/androgens**	↓	↓	↓	↓
**Free E2**	↑	→*		N
**Testosterone**	↑	↑	↓	↓
**Free Testosterone**	→		↓	N
**Androgen conversion of estrone**	↑	↑		
**Progesterone**	↑	↓ or →		↓ or →

*There are other accounts that free E2 actually does elevate in women in autoimmune disease; what is not disputed, though, is the overall steroid hormone homeostasis disruption.

(Abbreviated and reprinted with permissions from Oxford Academic (100). N, normal; ↑, increase in that hormone; →, no change; ↓, decrease in that hormone.

While not strong enough to count as independent evidence, there are some TED statistics that could be explained by sex steroid involvement. For example, risk for TED is equal in pre-pubescent children – but shifts to 4:1 female to male odds after puberty (101).

Though less frequent in men, TED in men tends to be more severe (102). This is similar to SSc, (79) and sex steroid hormones participation this could be manifold:

1. Men have more testosterone which can be converted to estrogen *via* the IL-6 induced aromatase activity (34, 77, 79);
2. Men lack sufficient progesterone to oppose estrogen, and what little progesterone they have can be inhibited by IGF (103);
3. The baseline testosterone/estrogen imbalances coupled with the increased aromatization, may increase men’s E2 levels to significant levels, as happens in SSc. In SSc, male patients with scleroderma have considerably higher levels of E2 (average, 30.6 pg/mL) than both healthy men (average, 12.9 pg/mL) *and* postmenopausal women with the disease (24.2 pg/mL). Furthermore, the higher the E2 levels in these men, the higher the mortality rate. It appears that a large portion of testosterone in these patients undergoes aromatization. Of note, post-menopausal women with SSc also have higher E2 levels than healthy post-menopausal women (79).

There are glaringly few case reports in the literature that have examined this possibility. But one is notable. Øgard et al. observed a case where TED manifested in a 56-year-old postmenopausal female, who had no previous history of thyroid disease. She had been a heavy smoker (20/day), so her orbital tissues were primed with oxidative stress. She was given hormone replacement therapy to prevent osteoporosis and within a month she developed TED, with characteristic diplopia and lid retraction. She was then taken off the HRT and treated with corticosteroids. 6 years later, she was placed back on hormone replacement therapy, and again, developed TED. This time the patient refused steroids based on previous adverse events and the TED remitted after 10 months. They also mention a previous, similar case known to them, but did not publish the details (104).

Another case demonstrates a key piece of information for further inquires, that ERα is expressed in orbital fibroblasts. Cury et. al., obtained orbital fibroblasts during orbital decompression surgery from a female patient with inactive moderately severe-to-severe TED. They confirmed ERα expression with rtPCR. They treated the cells with dexamethasone (DEX) and the ERα expression was affected in a dose dependent manner. In the cells treated with 10 nM and 100 nM DEX, ERα gene expression was, respectively, 85% higher and 74% lower, than in the control group (105). Their *in vitro* data are not conclusive but suggest treatment of TED with glucocorticoids may be partially mediated through ERαs, which is not the first time that has been examined (105–107). Glucocorticoid treatment works only in some patients with TED and often cause a rebound effect, worsening the TED. It’s a leap, but perhaps there is an estrogen marker to be found here about ideal responders?

A third, unpublished case was the impetus for this entire line of inquiry and literature review. I observed a unique case of moderate-to-severe TED which had two starts. This patient was diagnosed with Graves’s disease in April of 2017, a sequala to sepsis. June 15, 2017, she was diagnosed with mild thyroid eye disease. She had photophobia and dry eyes. These were entirely resolved with a week of steroid eye drops; she had no further symptoms. Her thyroid status was difficult to control even with methimazole and propranolol. In November of 2017, she came down with a *staphylococcus* infection (she still had cytopenias from the sepsis) and her TSI rose considerably (**Table 3**). Her surgeon noted that the moderate/severe TED likely began then. So did other symptoms. Coincident to this timing was menstrual abnormalities. She had one very long cycle, then concurrent with the appearance of TED, there was a reduction of her menstrual cycle length, which shortened from a regular 27-day cycle to a regular 13-day cycle, which usually indicates lack of progesterone. Every 10^th^ cycle day (CD), her TED signs and symptoms became noticeably worse, and then noticeably better at the start of her menses 4 days later (CD1). Self-described extreme premenstrual dysphoria also began at his same time, which was not in her previous history. After 6 short cycles, she then had three long cycles, and then menses arrested shortly after turning 45, young for her family by 6 years.

**Table 3:** Patient thyroid hormones.

	Reference range	4/27/2017	11/9/2017	2/12/2018	4/9/2018	7/11/2018	5/4/2019	9/8/2019
		Graves diagnosis	Staph infection	Moderate/severe TED diagnosis	P4 caused T4/3 increase; MMI increased	Eyes get much worse due to steroid rebound	Off MMI 10 months; TED stable 3 months	3 weeks post op
T4	0.8-1.8	2.04	2.9	2.5	3.9	1.6	1.4	1.1
T3	2.3-4.2	5.17	5.3	4.1	7.7	3.1	2.9	2.3
TSH	.4 – 4.5	<.003	0.01	<.01	<.01	<0.01	<0.01	1.42
TSI Ab	<140	343	582	578	543	506	542	498

AB, antibody; MMI, methimazole; P4, progesterone; TSI thyroid stimulating immunoglobulin.

Green, in range; red, out of range.

Her TED signs and symptoms ebbed and flared in synchrony with the estrogen valleys and peaks of her menstrual cycle, every cycle getting a bit worse. It was not a linear progression. The worst inflammation, edema, dryness, pain, and lid retraction always corresponded to the highest estrogen peak. The highest estrogen levels in a menstrual cycle are in the days just preceding ovulation. Luteinizing hormone tests are a proxy for ovulation timing, and she self-administered these on cycle day (CD) 10 to ensure the effect. These tests administered again on CD 3 of the following cycle to ensure luteinizing hormone was not high the whole cycle.

When her cycles lengthened, she would get a second flare corresponding to the 2^nd^ estrogen peak. She did have some relief and reduction in swelling with high dose progesterone prescribed by her gynecologist, but that had to be discontinued as it increased her T4 considerably (**Table 3**). In May 2018 she was given a 10-week course of high dose IV methylprednisolone, which temporarily reduced proptosis 1-2 mm, but had a rebound effect and the eye disease became much worse by August. Once the proptosis stabilized it was mainly the lid retraction that ebbed and flowed with her cycle. By the Spring of 2019, her thyroid had been sufficiently damaged by the TSI and by summer she was hypothyroid and entering menopause. She had bilateral orbital decompression and blepharoplasty in August 2019. The lid retraction persisted, and she had a second and final surgery November 2020.

Her estrogen levels throughout were at the upper limit of normal or higher than normal. Her progesterone and testosterone were low, though with as much progesterone as she was taking, it’s impossible to get an accurate read out **(** **Table 4** **).**

**Table 4:** Patient steroid hormones.

	Reference ranges*		
		**4/10/2018 - CD12 – Follicular phase**	
Estradiol	19-144 pg/mL	122	Both are quite near to the upper limit of normal for a 44-year-old woman
Total serum estrogens	90-590 pg/mL	552.3	
Progesterone	< 1.0 ng/mL	1.2	Elevated because she was taking 300 mg progesterone/night x1 month
Free testosterone	0.1-6.4 pg/mL	0.4	
		**8/11/2018 CD 26 – Luteal phase**	
Estradiol	56-214 pg/mL	96	
Progesterone	2.6-21.5 ng/mL	7.1	Possibly still high from previous progesterone intake
Free testosterone	0.1-6.4 pg/mL	0.6	
		**8/6/2019 Menopausal**	
Estradiol	< or = 31 pg/mL	81	Her last menses was 9/2018, but her estradiol remains above normal limits
Progesterone	< 0.5 ng/mL	0.2	Taking 200mg progesterone/night
Free testosterone	0.1-6.4 pg/mL	0.6	

*Reference ranges vary during the follicular & luteal phases of the menstrual cycle.

**Figure 3:**
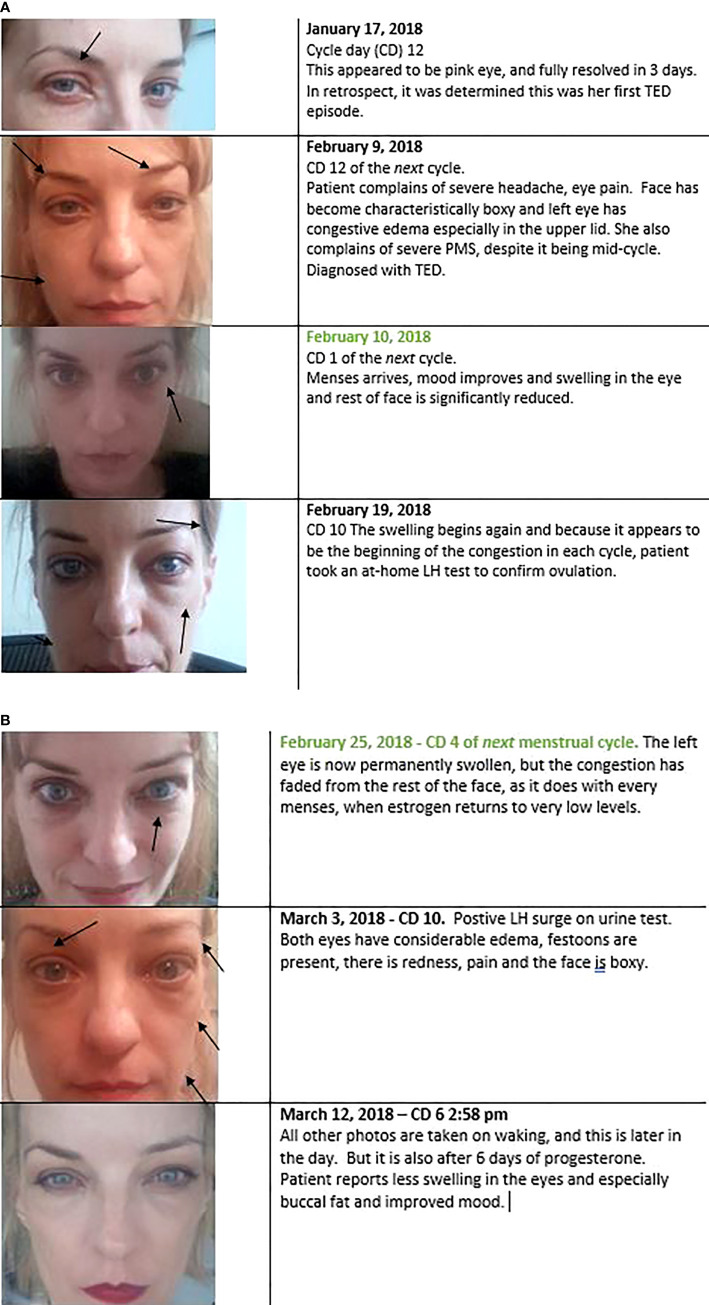
**(A, B)**. Every preovulation or premenstrual period, during concomitant estrogen peaks, patient’s TED signs and symptoms would become dramatically worse. Typically, this started on cycle day 10. Her cycles were short when the TED started (13 days total, indicating a possible lack of progesterone). When menses came and estrogen levels dropped to near zero (**Figure 5**), periorbital congestion and edema also dropped dramatically. This is evident in the one-day change photographed in the 2^nd^ and 3^rd^ panels. This was not edema-specific, it also occurred in the eyelid retraction, even after bilateral orbital decompression (not shown here). The lids retracted 1-2mm above the iris from cycle days 9 – 13, and then lowered to the level of the iris from cycle days 1 – 4.

Regrettably, as I am not an oculoplastic surgeon, I do not have CAS scores for her disease, but we have the below visual diary **(** **Figures 3A, B** **)**. All photos were taken in the morning, upon waking, except where noted. It is important to show the whole face, as TED also affects the buccal fat and gives the whole face a boxy appearance.

The author is well aware that this is far from definitive evidence. The paucity of direct evidence of estrogen’s role in TED does not mean there is no connection – merely that this has not been an area of investigation. This is curious because of all these autoimmune diseases; TED is itself borne out of endocrine disorders. Why has participation of the sex steroid hormones not been thoroughly evaluated in TED when they play clear roles in autoimmune diseases that do not have this underlying hormone dysregulation?

Finally, there is one other area that estrogen research into TED could prove particularly intriguing: the relationship of estrogen and growth hormones. While the below is also not direct evidence, it is further rationale for speculation and investigation.

## IGF-1R Has Significant Cross Talk With ERs and E2

IGF-1R is instrumental to TED pathophysiology. For unknown reasons, orbital fibroblasts in patients with TED express higher than normal numbers of IGF-1Rs (1). There is currently a debate in the literature about the presence of an IGF-1R autoantibody in Graves’s disease, but all agree that TSHR once bound by an autoantibody, affixes to IGF-1R *via* β-arrestin and IGF-1R is necessary for the ensuing inflammatory cascade (4, 108).

**Figure 4:**
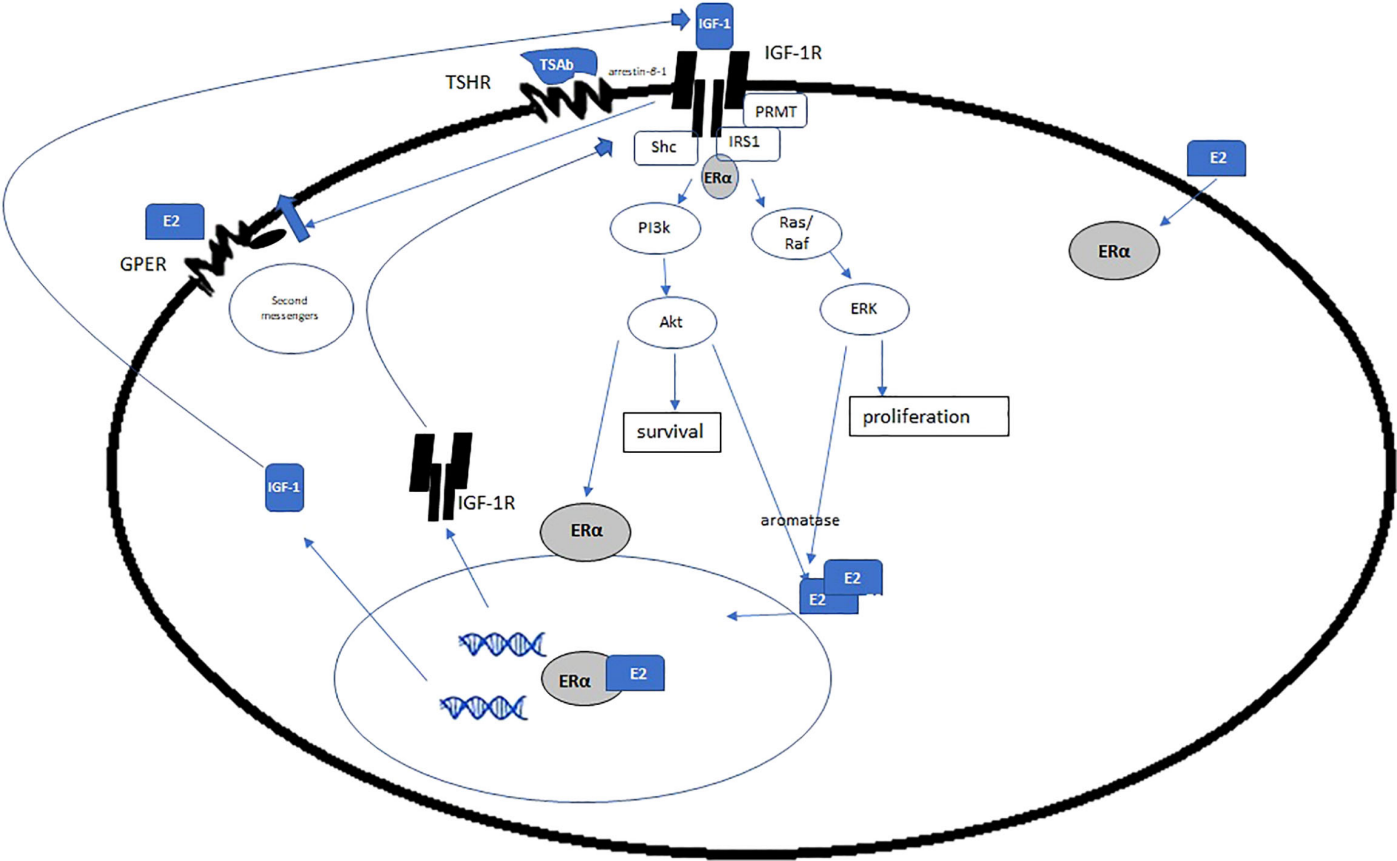
Estrogen and ERα exert genomic and non-genomic effects on IGF-1R, IGF, and other growth factors. Given the increase in E2 in GD and HD, is there a concomitant increase in ERα and IGF-1R, via the mechanisms posited here? If so, it is plausible that estrogen is a disease driver in TED. (Adapted with open access creative commons permission from 115).

Steroid hormones, luteinizing hormone and growth factors regulate expression of IGF-1R (109, 110). E2 and estrogen receptors, specifically, have been shown to modulate IGF-1R in a variety of tissue types and diseases in both animal and human models (111, 112). ERα knock out murine models lower serum levels of IGF-1 compared with their intact ERα^+^ littermates (113). In breast cancer, in model autoimmune diseases, as in TED, the binding of IGF-1 to IGF-1R triggers the PI3K/AKT/mTOR signaling pathway (17–20, 114, 115). In breast cancer, then a downstream molecule of the AKT/mTOR pathway phosphorylates ERα which binds to promoter of target genes, and upregulates IGF-1, IGF-1R (115, 116) (**Figure 4**). Estrogen also stimulates cytosolic ER to interact with adaptor proteins, such as Shc or the p85 subunit of PI3K. This complex then translocates to the membrane where it associates with IGF-1R leading to activation of downstream signaling cascades like MAPK (117, 118).

ERα rapidly activates IGF-1R in non-carcinogenic cells, as well. IGF-1R and ERα colocalize in in many cell lines (119). Kahlert et al. demonstrated that E2 *via* ERα, is capable of IGF-1R phosphorylation and that these effects could be blocked with estrogen antagonists. They further showed that constitutively active MAPKK induced an interaction of ERα with the IGF-1R even in the absence of ligand. Their in-depth analysis of these interactions concludes that ***“*** taken together, these data demonstrate that ligand bound estrogen receptor α is required for rapid activation of the IGF-1R signaling cascade” (120).

Oesterreich et al. showed that ERα is a pivotal regulator of IGF mitogenesis. They used MCF-7 breast cancer cells which had lost their ERα and showed reduced expression of IGF-signaling and failure to proliferate when stimulated with E2 or IGF-1. Transection-facilitated re-expression of ERα restored the IGF-1-responsives and proliferation (121).

Chan et al., in a murine breast cancer model found that E2 significantly increased basal phosphorylation of IGF-1R, IRS-1, IRS-2, and Akt-1, but that basal phospho p44/42 MAPK was significantly reduced. These effects were extinguished with the anti-estrogen fulvestrant. Furthermore, high doses of fulvestrant increased decreased IGF-1R and its autophosphorylation to approximately 30% of the controls. IRS-1, IRS-2, and c-Raf-1 levels were similarly reduced.

The PI3K pathway is instrumental to cellular metabolism, affording the subsequent development, survival, and proliferation of the cell. For example, PI3Kδ regulates B cell ability to develop from transitional to naïve to plasmablast to antibody secreting plasma cell, and even to undergo class switch recombination (48, 122). Estrogen, *via* its nuclear and membrane receptors, and IGF-1 complex, and the PI3K pathway can affect gene transcription that affords the cells the metabolic requirements for development (123–126).

Another example of can be found in uterine fibroids (leiomyomas) (112, 127). IGF-1 expression is most abundant in leiomyomas during the proliferative, estrogen dominated phase of the menstrual cycle. Yu et al. have shown that estrogen upregulates the gene encoding IGF-1 through ERα in leiomyoma tissue and cells during this time (112). Finally, estrogen also has direct and rapid effect on growth hormone, which also exerts effects on IGF-1 (128, 129).

## Growth Hormone, IGF-1 and Estrogen

**Figure 5:**
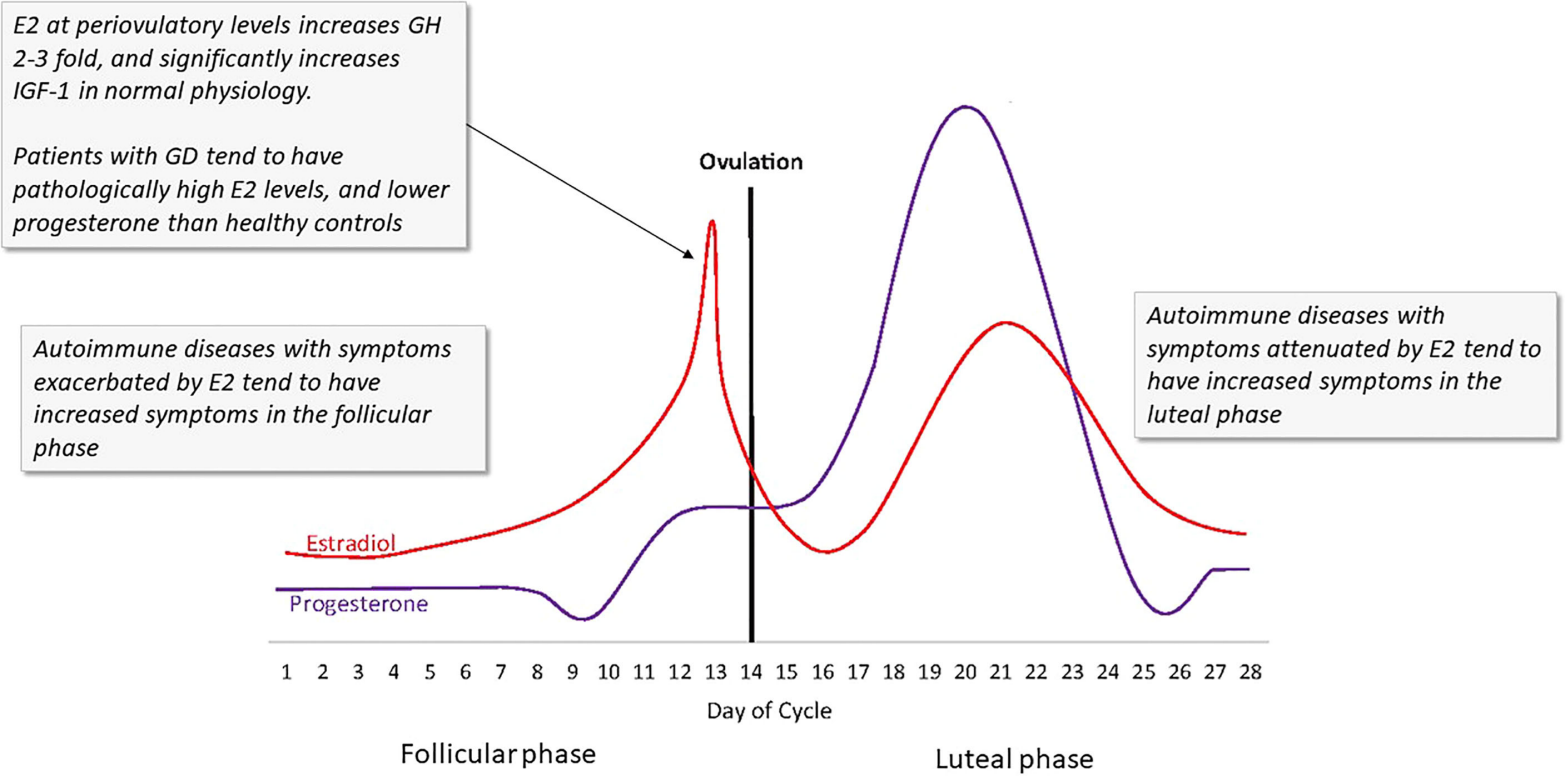
The ebb and flow of steroid hormones in a typical menstrual cycle. Several autoimmune disease symptoms are exacerbated by the rises in estrogen seen in both the first (follicular) and second (luteal) phases of the cycle. In GD and HD, these effects could be more profound due to the lack of steroid hormone homeostasis. Estrogen at periovulatory and premenstrual levels significantly increases IGF-1R, IGF, and GH. (Image copied with permission from 131).

Finally, estrogen can regulate IGF *via* growth hormone (GH). Estrogen has direct and rapid effect on GH (128, 129). At periovulatory levels in healthy women, E2 can increase GH 2-3-fold (129, 130). In that same periovulatory period in healthy women, serum IGF-1 levels (though not its binding proteins) are significantly increased. That measurement is of physiological levels of estrogen, not at the ~2-3x higher, pathological levels seen in GD (95, 100). Consequently, at periovulatory levels in GD of HD, the GH/IGF-1 increase during periovulatory days may be considerable **(** **Figure 5** **)**.

## Stromal Metabolism and Estrogen

As noted, fibroblasts, which fall into the larger class of stromal cells, are key to the homeostasis of the tissues they reside on and can function as sentinels. Their own metabolism, and the metabolism of their microenvironment can be influenced by E2. In a series of breast cancer murine model experiments, Péqueux et al, demonstrated that *via* the ERα microenvironment of stromal cells, E2 can facilitate tumor growth by triggering stromal cells, increasing angiogenesis, improving vessel morphology and architecture (facilitating durable metastasis) and reduced tumor hypoxia. They conclude because of these changes in the vessels, stromal ERα is a necessary component of tumor growth, as it improves oxygen and nutrient delivery, preventing hypoxia and necrosis (132). Specific fibroblast growth factors which mediate angiogenesis and mitogenesis in the stromal cells of endometriosis disease models have also been found to be dependent on aberrant production of E2 and these effects can be extinguished with an aromatase inhibitor (133). Given E2’s direct effects on stromal cells and given its regulation of myriad growth factors, and the consequence of these factors on stromal calls ability to sustain aberrant growth, it would seem likely that in a stromal tissue disease dependent on growth factors, such as TED, that the steroid hormones play a role.

## Discussion

**Figure 6:**
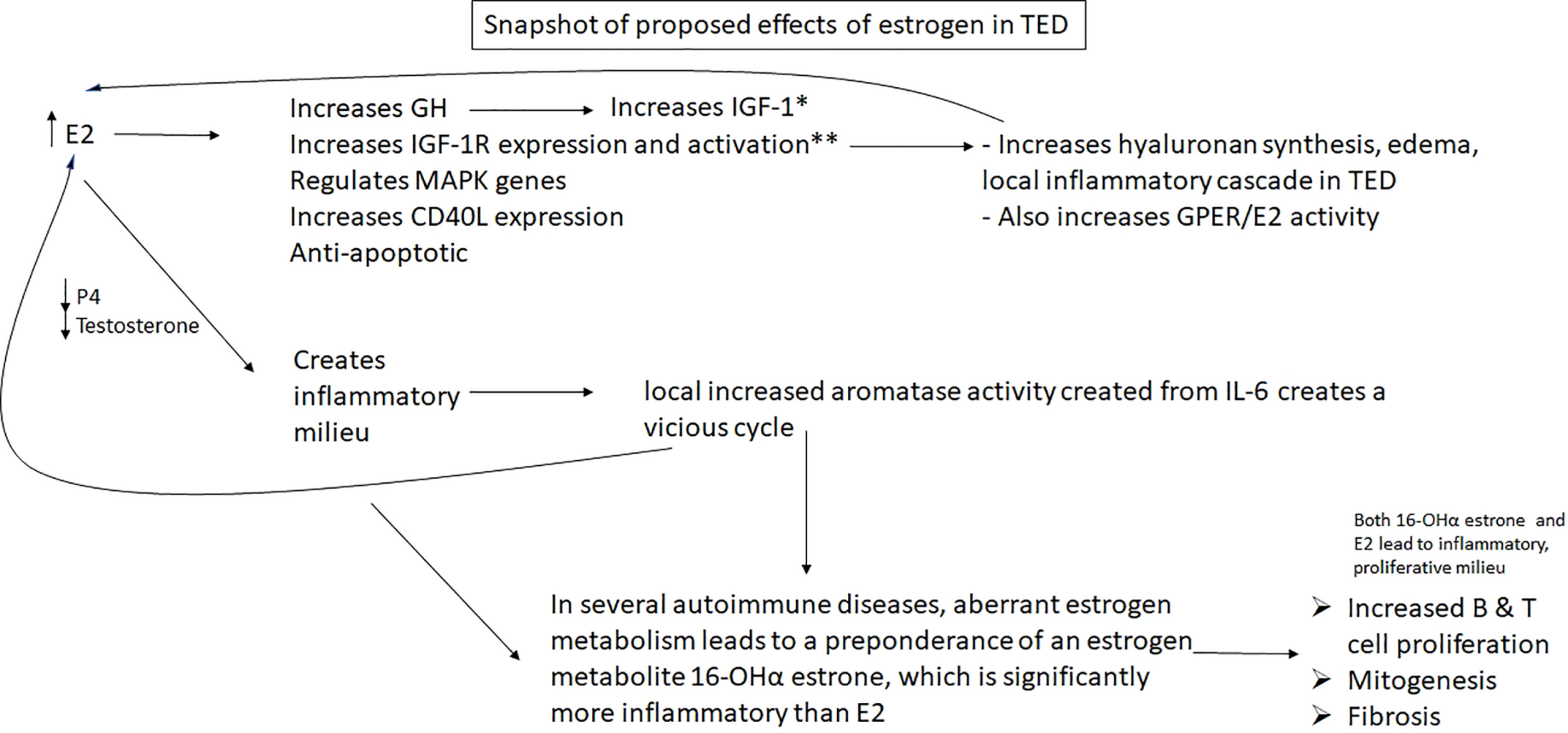
Hypothesis: Excess estradiol and reduced progesterone & testosterone seen in male and female patients with Graves’ and Hashimoto’s disease may increase growth factors and IGF-1 receptors, helping to facilitate the well characterized TED inflammatory pathway elicited by IGF-1R; it also may result in a vicious cycle with local IL-6, which will create more E2 by aromatizing local androgens, which will stimulate the creation of more IL-6, in addition to other downstream inflammatory effects. E2, estradiol; ERα, estrogen receptor α; IGF-1R, insulin-like growth factor 1 receptor; GH, growth hormone; P4, progesterone; *E2 has varying direct effects on IGF-1 depending on age, gender, concentration; **Luteinizing hormone, which is also increased in women in Graves’ disease, meaningfully increases IGF-1R as well.

There are two large questions in TED that remain enigmatic. *1) Why does euthyroid status matter?* What is protective about having thyroid hormone levels within normal limits because that status does not mean the person has low autoantibodies. But those autoantibodies remain in contact with the thyroid, not the orbit more often if they person is euthyroid. The persistence of either hypo- or hyperthyroidism correlates with increased severity of TED (134). What happens when someone remains hyper- or hypothyroid? *Other* hormones also become dysregulated. Sex steroid hormones, which become meaningfully dysregulated, all influence inflammation, fibrosis, and growth factors. It is my contention that sex steroid homeostasis has the potential to contribute meaningfully to TED, and this should be investigated (**Figure 6**). If there is merit to this hypothesis, it could explain the importance of euthyroid status on development and severity of TED and might even yield a biomarker. It would also shed light on why TED has been reported to be worse in post-menopausal women and in men.

*Question 2 is*, *why is IGF-1R is overexpressed in fibroblasts of patients with TED?* Sex steroid homeostasis is disrupted, skewed toward high estrogen in patients with uncontrolled GD or HD, which are the preponderance of people who get TED. Estrogen regulates IGF-1R and growth hormones. The impetus to investigate if estrogen has contributed to this overexpression or pathology with the growth hormone and its receptor seems axiomatic (**Figures 4**, **5**).

Though the above are speculative, there are corollaries that could serve as models. The type of inflammation and fibrosis seen in TED are not unique. Pathological IGF-1R/IGF-1 mediated tissue proliferation as seen in TED is also not unique. It is seen in breast cancer and uterine leiomyomas. Estrogen is pivotal to all of these pathologies; it certainly could be in TED. As with any investigation in science, these queries into the sex steroid hormones in TED may not yield a theory, or it may bloom in unexpected directions. That happened with rheumatoid arthritis, when unexpected subtypes were revealed based on estrogen pathology – and this shed light on the variance of responses to RA treatments (27).

The extensive and careful investigations on the role of estrogen, estrogen receptors and estrogen metabolites in autoimmunity conducted in RA, SSc and SLE bear repeating in the orbital fibroblasts of patients with thyroid eye disease, as do examinations of estrogen’s effects on IGF-1R. If transitive properties do exist, it will not only shed light on TED, but open a door to further considering IGF-1R’s possible role in these other 3 AIDs – a surface already scratched by Drs. Douglas and Smith in 2010 (135). There are many lines of inquiry possible here, a few initial ones are:

• Orbital fibroblasts (OF) express ERα (105). Does bathing them in E2 modify IGF/IGF1R expression?
   ○ If yes, can this be extinguished or attenuated with fulvestrant? With progesterone?
      ▪ Does increased IGF-1R increase ERα activity, engendering a vicious cycle?
   ○ Does E2 bathing increase expression of its own receptors? Often in the endocrine system, the reverse would happen, but there are examples of ligand saturation increasing its own receptors in the literature.
• Do OFs express GPER and/or ERβ?
   ○ Does the former participate in any IGF modulation?
   ○ Are the latter in too low a concentration?
• Are any three of the estrogen receptor types functionally polymorphic compared to healthy controls?
• Do patients with TED have a preponderance of inflammatory estrogen metabolites?
  ○ This is a simple urine test, and could be examined in healthy controls, patients with GD or HD without TED, and in patients with TED
      ▪ If yes, do OFs bathed in 16αOH‐estrone, cause any IGF/IGF-1R changes? Does that metabolite bind covalently in these cases?
      ▪ Do these patients have variants in their cytochrome P450 gene aromatase enzymes (genetic test)
      ▪ If any of these metabolite studies yield data, could that become a biomarker?
• The unpublished patient case discussed above has high 4OH-estradiol, which is inflammatory, and low 2OH-estradiol; her sister who has Graves’ disease but not TED is getting her estrogen metabolism evaluated

TED occurs almost exclusively in patients with endocrine hormone disorder. Investigating the endocrine system in these patients is an as yet unexplored arena. Based on the above speculations, it could be worthy of exploration.

This review is far from an exhaustive survey on the sex steroid hormones’ influence on the immune system. Interested readers are directed to references for exhaustive findings of this complex interaction (27, 32, 34, 49, 87, 136).

## Data Availability Statement

The original contributions presented in the study are included in the article/supplementary material. Further inquiries can be directed to the corresponding author.

## Ethics Statement

Written informed consent was obtained from the individual(s) for the publication of any potentially identifiable images or data included in this article.

## Author Contributions

The author confirms being the sole contributor of this work and has approved it for publication.

## Funding

All research was funded and conducted solely by the author.

## Conflict of Interest

The author declares that the research was conducted in the absence of any commercial or financial relationships that could be construed as a potential conflict of interest.

## Publisher’s Note

All claims expressed in this article are solely those of the authors and do not necessarily represent those of their affiliated organizations, or those of the publisher, the editors and the reviewers. Any product that may be evaluated in this article, or claim that may be made by its manufacturer, is not guaranteed or endorsed by the publisher.
